# Desenvolvimento de modelo clínico para predição da possibilidade de identificação da artéria de Adamkiewicz por angiotomografia

**DOI:** 10.1590/1677-5449.006317

**Published:** 2018

**Authors:** Alexandre Campos Moraes Amato, José Rodrigues Parga, Noedir Antônio Groppo Stolf

**Affiliations:** 1 Instituto de Medicina Avançada – AMATO, Cirurgia Vascular e Endovascular, São Paulo, SP, Brasil.; 2 Universidade de Santo Amaro – UNISA, Disciplina de Cirurgia Vascular, São Paulo, SP, Brasil.; 3 Universidade de São Paulo – USP, Departamento de Radiologia, Faculdade de Medicina, Hospital das Clínicas, Instituto do Coração, São Paulo, SP, Brasil.; 4 Universidade de São Paulo – USP, Cirurgia Torácica e Cardiovascular, São Paulo, SP, Brasil.

**Keywords:** medula espinhal, coluna vertebral, aorta, Adamkiewicz

## Abstract

**Contexto:**

Diferenças morfológicas da artéria de Adamkiewicz (AKA) entre a população portadora e não portadora de doença aórtica têm importância clínica, influenciando as complicações neuroisquêmicas da medula espinhal em procedimentos operatórios. Ainda não é conhecida a correlação entre parâmetros clínicos e a previsibilidade da identificação dessa artéria pela angiotomografia.

**Objetivo:**

Desenvolver um modelo matemático que, através de parâmetros clínicos correlacionados com aterosclerose, possa prever a probabilidade de identificação da AKA em pacientes submetidos a angiotomografias.

**Método:**

Estudo observacional transversal utilizando banco de imagens e dados de pacientes. Foi feita análise estatística multivariada e criado modelo matemático logit de predição para identificação da AKA. Variáveis significativas foram utilizadas na montagem da fórmula para cálculo da probabilidade de identificação. O modelo foi calibrado, e a discriminação foi avaliada pela curva *receiver operating characteristic* (ROC). A seleção das variáveis explanatórias foi guiada pela maior área na curva ROC (p = 0,041) e pela significância combinada das variáveis.

**Resultados:**

Foram avaliados 110 casos (54,5% do sexo masculino, com idade média de 60,97 anos e etnia com coeficiente B -2,471, M -1,297, N -0,971), com AKA identificada em 60,9%. Índice de massa corporal: 27,06 ± 0,98 (coef. -0,101); fumantes: 55,5% (coef. -1,614/-1,439); diabéticos: 13,6%; hipertensos: 65,5% (coef. -1,469); dislipidêmicos: 58,2%; aneurisma aórtico: 38,2%; dissecção aórtica: 12,7%; e trombo mural: 24,5%. Constante de 6,262. Fórmula para cálculo da probabilidade de detecção: ${\left(e^{-\left(Coef.\;Etnia+\left(Coef.\;IMC\times IMC\right)+Coef.fumante+Coef.HAS+Coef.dislip+Cons\tan te\right)}+1\right)}^{-1}$ . O modelo de predição foi criado e disponibilizado no link https://vascular.pro/aka-model  .

**Conclusão:**

Com as covariáveis etnia, índice de massa corporal, tabagismo, hipertensão arterial e dislipidemia, foi possível criar um modelo matemático de predição de identificação da AKA com significância combinada de nove coeficientes (p = 0,042).

## Introdução

 O estudo das diferenças morfológicas da artéria de Adamkiewicz (AKA) entre a população de portadores e não portadores de doença tem importância clínica, pois pode influenciar as complicações neuroisquêmicas da medula espinhal em procedimentos operatórios. Pode também auxiliar a estabelecer a importância de se preservar essa artéria em cirurgias aórticas e neurológicas, diminuindo o risco de isquemia medular 1 ^-^ 3 . 

 As artérias intercostais e lombares que alimentam a medula espinhal originam-se da aorta. As artérias lombares e intercostais dividem-se três vezes antes de alcançar a medula espinhal. A última bifurcação do ramo espinhal é constante para o suprimento anterior e posterior do canal vertebral, somente em alguns níveis. Geralmente, uma das artérias radiculares anteriores é dominante perante as outras em calibre e é chamada de artéria radicular anterior magna ou AKA 2 . 

**Figura 1:**
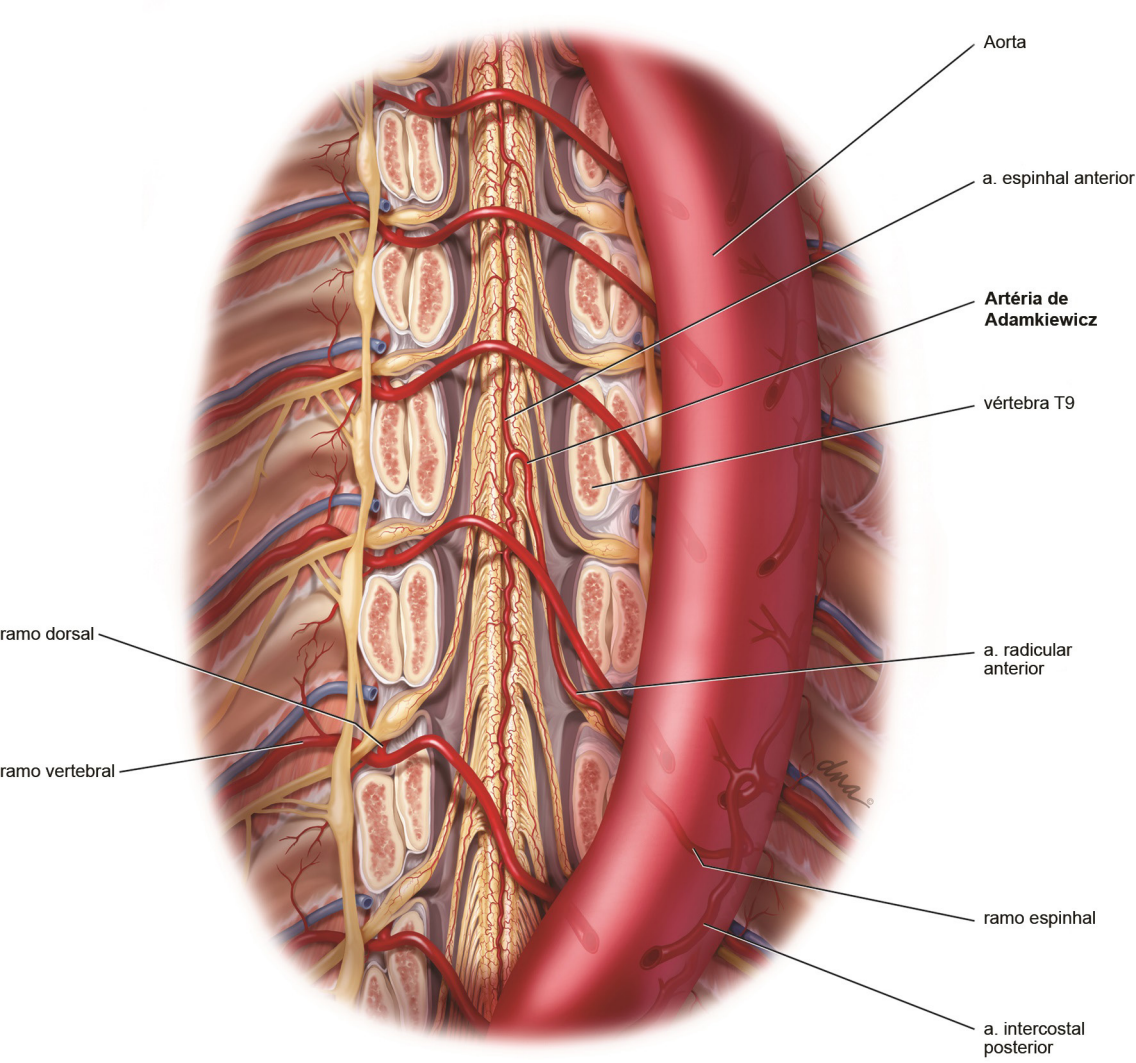
Representação anatômica ilustrada da irrigação medular com remoção do corpo vertebral anteriormente.

 O conhecimento da irrigação da medula espinhal é importante no planejamento terapêutico das doenças aórticas; porém, sua vasculatura é complexa e difícil de estudar devido ao pequeno calibre de suas artérias, que correm em uma intrincada rede tridimensional com grande variabilidade anatômica 2 ^,^ 4 ( Figura 1 ). A ausência de um consenso sobre o exame de imagem padrão-ouro também dificulta a comparação dos métodos de imagem existentes. 

 As diferenças na importância da AKA na irrigação medular entre as doenças, como aneurismas, dissecções, presença de trombo mural e outras, também não são conhecidas, embora essa seja a população mais propensa à necessidade de tratamento cirúrgico e, consequentemente, mais exposta ao risco de isquemia medular. Foi demonstrado que a própria doença aórtica pode alterar a visualização da irrigação medular 5 . Apesar do conhecimento dos fatores que influenciam a identificação da irrigação medular na angiotomografia, o efeito concomitante deles não é conhecido. 

## Objetivo

 Criar um modelo matemático de predição para a identificação ou não da AKA na angiotomografia utilizando variáveis clínicas. 

## Método

### Casuística

 Realizamos um estudo observacional transversal envolvendo banco de dados e imagens de estudo prévio 5 em pacientes portadores ou não de doença aórtica, submetidos a angiotomografia entre outubro de 2011 e julho de 2012. 

 Nesse período, no Instituto do Coração do Hospital das Clínicas da Faculdade de Medicina da Universidade de São Paulo (InCor-HC-FM-USP), foram realizadas 128 angiotomografias de aorta no equipamento Aquilion One (Toshiba™, Medical Systems, Otawara, Japão) no departamento de diagnóstico por imagem. Dessas, 110 foram consideradas elegíveis, com base nos critérios de inclusão e exclusão. 

 Os critérios de inclusão utilizados foram os seguintes: pacientes que realizaram angiotomografia de aorta no equipamento Aquilion One seguindo protocolo predefinido, independentemente do motivo da realização do exame; pacientes que aceitaram a realização da angiotomografia de aorta, caso não tivesse sido solicitada pelo médico de origem, como complemento do exame. 

 Os critérios de exclusão utilizados foram: pacientes que realizaram cirurgia prévia de aorta descendente; pacientes portadores de doenças incomuns que pudessem comprometer os resultados devido à presença de circulação colateral (Takayasu, coarctação aórtica); pacientes paraplégicos ou tetraplégicos; alergia conhecida a contraste radiológico; inclusão concomitante em outro estudo que interferisse no protocolo; pacientes com hematoma intramural e úlcera penetrante aórtica; idade menor que 25 anos para homogeneidade da amostra; falha técnica na opacificação da aorta. 

 Procedeu-se à análise prospectiva de angiotomografias realizadas em pacientes de rotina em ambulatório, por meio do *software open source* OsiriX *®* (Pixmeo, Genebra, Suíça) 6 . A análise das imagens foi cega; o avaliador não tinha conhecimento do motivo da realização do exame e a avaliação da presença de doença aórtica somente foi realizada em um segundo ciclo de avaliação das imagens, também sem conhecimento da identificação ou não da AKA. 

 A angiotomografia seguiu o padrão preestabelecido: as imagens foram adquiridas no mesmo aparelho Aquilion One de 320 detectores, com *software* para detecção helicoidal com 80 a 160 detectores, seguindo protocolo de fase arterial contrastada por 100 a 110 mL de contraste não iônico tri-iodado administrado por via intravenosa em 30 s com bomba injetora, na velocidade de 4 a 5 mL/s, com gatilho configurado para maior concentração de contraste em aorta descendente com limiar (*threshold*) de 150 HU. Os parâmetros técnicos do método angiotomográfico seguiram a recomendação das diretrizes de diagnóstico e tratamento de doenças aórticas 7 . 

 Foi realizada a estratificação das seguintes características dos pacientes: sexo, idade, grupo étnico, peso, altura e índice de massa corporal (IMC). A presença ou não da artéria espinhal anterior, a presença e localização da AKA e a presença ou não de doença aórtica, trombo e dissecção foram avaliadas por observador único e experiente diretamente nas imagens de maior qualidade *raw* DICOM do exame do paciente. Secundariamente, foram avaliados os fatores de risco dicotômicos relacionados às doenças aórticas: diabetes melito, síndrome metabólica, hiperlipidemia e hipertensão arterial. A variável tabagismo foi avaliada como fumante, ex-fumante e não fumante. 

 Os dados sociodemográficos foram obtidos nos prontuários e por contato direto com os pacientes e seus familiares: idade, sexo e etnia. A etnia foi definida pela cor relatada pelo participante e considerada autoavaliação. Variações multirraciais autodeclaradas, como mulato e mestiço, foram entendidas como pardo. Permitiu-se ignorar a pergunta. 

 A coleta dos dados se deu a partir da anamnese dos pacientes, de informações obtidas nos prontuários e por contato telefônico com familiares quando necessário. Os seguintes parâmetros foram avaliados: peso, altura e IMC. Peso e altura foram aferidos em balança antropométrica; o IMC foi calculado pela fórmula: peso (em quilogramas) dividido por altura elevada ao quadrado (em metros). Tabagismo: considerou-se tabagista atual o indivíduo que fumou um ou mais cigarros nos últimos 30 dias e ex-tabagista quem fumou há mais de 30 dias. Diabetes melito (DM): foram considerados diabéticos aqueles que faziam uso prévio de hipoglicemiante oral e/ou insulina, ou com duas dosagens de glicemia de jejum ≥ 126 mg/dL. Hipertensão arterial sistêmica (HAS): foram considerados hipertensos pacientes em uso de anti-hipertensivo e aqueles com pressão arterial sistólica (PAS) acima de 140 mmHg e/ou pressão arterial diastólica (PAD) acima de 90 mmHg. Dislipidemia: foram considerados dislipidêmicos aqueles pacientes em uso de hipolipemiantes e/ou elevação de colesterol de lipoproteína de baixa densidade (LDL-C ≥ 160 mg/dL) e/ou elevação de triglicérides (TG ≥ 150 mg/dL) e/ou colesterol de lipoproteína de alta densidade (HDL-C) < 40 mg/dL para homens e < 50 mg/dL para mulheres 8 . Síndrome metabólica: o diagnóstico de síndrome metabólica foi estabelecido segundo critérios da Federação Internacional de Diabetes. Inclui a presença de obesidade abdominal como essencial e dois ou mais dos seguintes critérios: triglicérides ≥ 150 mg/dL; HDL-C < 40 mg/dL para homens e < 50 mg/dL para mulheres; PAS ≥ 130 mmHg e/ou PAD ≥ 85 mmHg ou tratamento para hipertensão; glicemia de jejum ≥ 100 mg/dL ou tratamento para diabetes melito 8 . 

 O método de identificação da AKA já foi utilizado pelo autor, publicado e validado previamente 3 ^,^ 6 , e o vídeo explicativo da técnica está disponibilizado em http://vascular.cc/aka.html  . 

### Análise estatística

 As informações obtidas a partir da anamnese e dos prontuários dos pacientes foram digitadas em um banco de dados *online* seguro (GoogleDocs®). Após a verificação da consistência dos dados, procedeu-se à análise descritiva de cada grupo de pacientes. 

 A análise estatística multivariada dos dados foi efetuada utilizando o *software* Wizard 1.9.7 (Evan Miller, Chicago, US). Área da curva característica de operação do receptor (*receiver operating characteristic* , ROC) maior que 0,7 foi considerada um bom modelo e maior que 0,85 foi considerada um excelente modelo. Optou-se pelo modelo logit pelo desfecho binário, buscando significância com p < 0,05. Após a criação do modelo, foi criada uma fórmula de predição para a variável de desfecho. A fórmula de predição calcula o valor esperado do desfecho (identificação ou não da AKA) baseada nas variáveis iniciais selecionadas que preencheram os critérios para a área da curva ROC maior que 0,7. 

**Figura 2:**
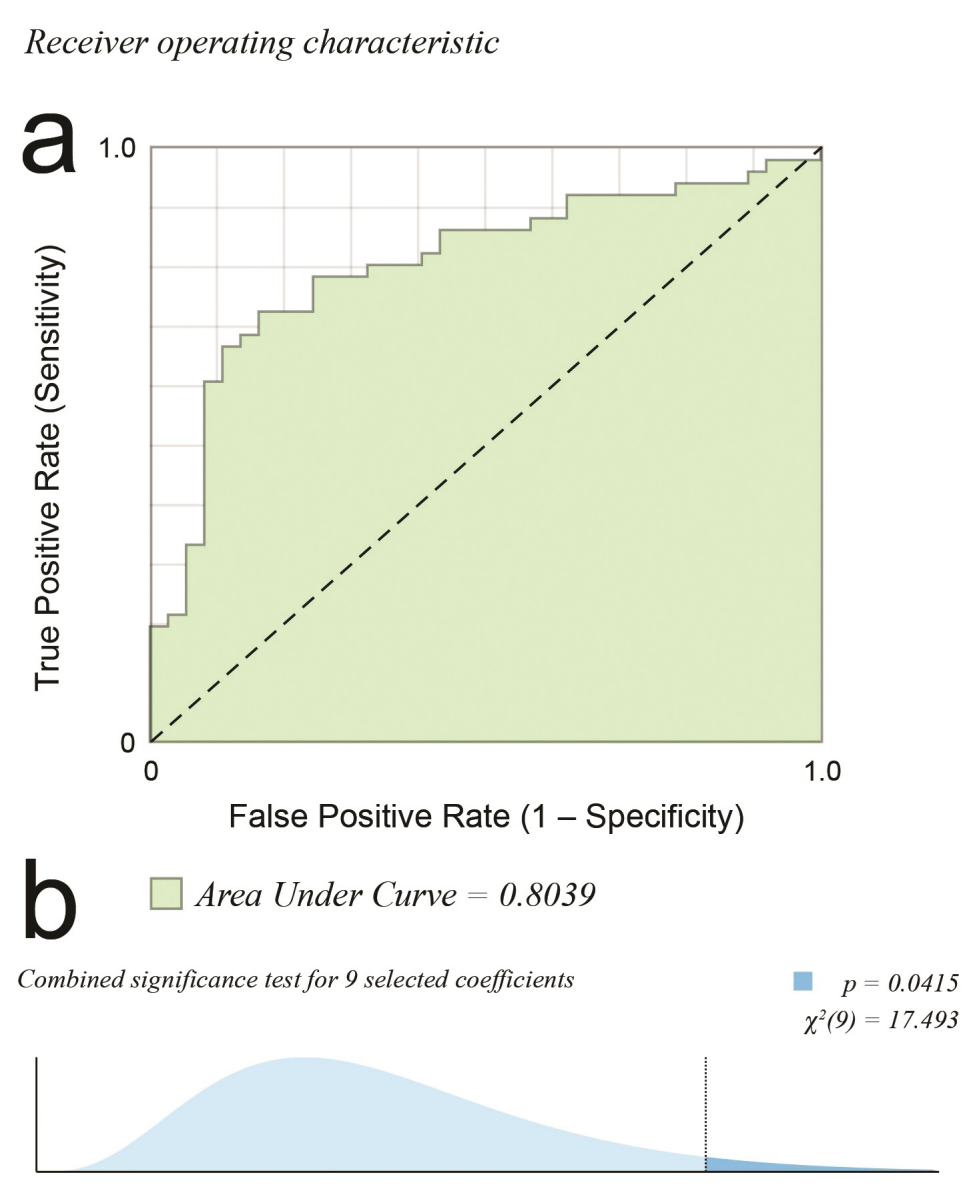
(a) Curva característica de operação do receptor (ROC). Quanto maior a área, melhor o ajuste (1,0 = perfeito; 0,5 = aleatório). Área sob a curva de 0,8039; (b) Distribuição t usada no teste da hipótese centrado no ponto da estimativa, com teste de significância para nove covariáveis selecionadas, resultando em p = 0,0415.

 Considerando a amostra global, a Tabela 1 lista as variáveis coletadas e as análises preliminares do banco de dados já publicados 3 ^,^ 5 . Foi selecionado como desfecho a identificação ou não da AKA pelo método proposto. O conhecimento prévio da influência da massa corporal, da doença arterial e da aterosclerose guiou a eleição inicial das covariáveis explanatórias. Foram mantidas as covariáveis com o coeficiente significativamente diferente de zero para o nível de 10% e, assim, etnia, tabagismo, IMC, hipertensão e dislipidemia foram escolhidas para a criação do modelo de predição ( Tabela 2 ). A seleção das variáveis explanatórias foi guiada pela maior área da curva ROC ( Figura 2 A) e pela significância combinada das variáveis ( Figura 2 B), de modo que o equilíbrio em número de variáveis explanatórias, sem excesso, e maior influência na ROC fosse atingida. 

**Tabela 1:** Dados sociodemográficos da população estudada.

**Característica**		**N = 110**
Sexo	Masculino	60 (54,5%)
	Feminino	50 (45,5%)
Idade	Média	60,97 ± 2,34
	
IMC	Média	27,06 ± 0,98
	
Etnia	Amarelo	6 (6,8%%)
	Branco	55 (62,5%)
	Pardo	21 (23,9%)
	Negro	6 (6,8%)
AKA	Sim Não	67 (60,9%) 43 (39,1%)
Fumante	Sim Não Ex	61 (55,5%) 32 (29,1%) 17 (15,5%)
Diabetes	Sim Não	15 (13,6%) 95 (86,4%)
HAS	Sim Não	72 (65,5%) 38 (34,5%)
Dislipidemia	Sim Não	46 (58,2%) 64 (41,8%)
Síndrome metabólica	Sim Não	25 (22,7%) 85 (77,3%)
Aneurisma de aorta	Sim Não	42 (38,2%) 68 (61,8%)
Dissecção de aorta	Sim Não	14 (12,7%) 96 (87,3%)
Trombo mural	Sim Não	27 (24,5%) 83 (75,5%)

AKA, artéria de Adamkiewicz; HAS, hipertensão arterial sistêmica; IMC, índice de massa corporal.

**Tabela 2:** Significância da variável no modelo estudado e coeficientes de influência das variáveis no desfecho.

***Variável explanatória***	***Coeficiente***	***Standard error***	***p***	***Significância***
*Etnia*			0,0677	*
*Branco*	-2,471	(1,264)	0,0505	*
*Mulato*	-1,297	(1,329)	0,3291	
*Negro*	-0,971	(1,597)	0,5433	
*IMC*	-0,101	(0,055)	0,0664	*
*Fumante*			0,0196	**
*Sim*	-1,614	(0,801)	0,0439	**
*Ex*	-1,439	(0,595)	0,0156	**
*HAS*	-1,469	(0,604)	0,0150	**
*Dislipidemia*	0,97	(0,555)	0,0806	*
*Constante*	6,262	(2,018)	0,0019	***

* Coeficiente significativamente diferente de zero no nível de 10%;

** Coeficiente significativamente diferente de zero no nível de 5%;

*** Coeficiente significativamente diferente de zero no nível de 1%.

HAS, hipertensão arterial sistêmica; IMC, índice de massa corporal.

 Essa investigação foi realizada de acordo com os princípios da Declaração de Helsinki. Este estudo foi aprovado pela Comissão Científica do Instituto do Coração (InCor) da Universidade de São Paulo e pelo Comitê de Ética e Pesquisa do HC-FMUSP em 05/05/2010 sob o número de referência 0089/10. Está registrado no Sistema Nacional de Informações sobre Ética em Pesquisa (SISNEP) envolvendo seres humanos da Comissão Nacional de Ética em Pesquisa (CONEP) sob o número 0088.0.015.000-10. Todos os pacientes ou responsáveis foram esclarecidos sobre o objetivo deste estudo e concordaram e assinaram o termo de consentimento livre e esclarecido, sendo que a atual análise dos dados estava contemplada na permissão. 

## Resultados

**Figura 3:**
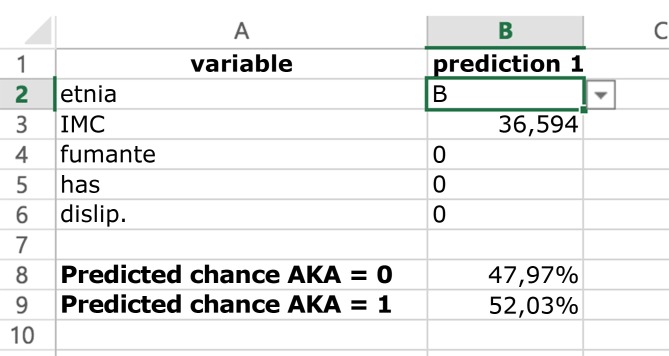
Aparência simplificada do modelo de predição de identificação da AKA em planilha eletrônica comum. Interface de coleta de dados para o cálculo da probabilidade do desfecho.

 A fórmula para cálculo da probabilidade de detecção da AKA ${\left(e^{-\left(Coef.\;Etnia+\left(Coef.\;IMC\times IMC\right)+Coef.fumante+Coef.HAS+Coef.dislip+Cons\tan te\right)}+1\right)}^{-1}$ permite a criação de modelo de predição criado e exportado para planilha Excel ( Figura 3 ). A constante e os coeficientes de influência das variáveis selecionadas para cálculo do desfecho são apresentados na Tabela 2 . Após formatação e envio para serviço *online* , o modelo foi publicado para utilização geral no *site* https://vascular.pro/aka-model  . As variáveis permitem a mudança em tempo real e calculam a probabilidade de identificação da AKA. 

## Discussão

 A isquemia medular é uma complicação da cirurgia aórtica que, apesar de não ser frequente, é devastadora e merece ser estudada 9 . O estudo *in vivo* da AKA é prejudicado, pois o método considerado padrão-ouro, a angiografia, apresenta complicações graves em uma frequência inaceitável atualmente: 8,2% para complicações locais, 3,7% para não neurológicas e 2,2% para neurológicas 10 , além de não apresentar detecção de 100% dos casos (sem doença aórtica: 68% 10 ; com doença aórtica: 60% 11 ). 

 As características angiotomográficas da AKA na população não portadora de doença aórtica e portadora de doença aórtica foi estudada previamente pelo autor 3 ^,^ 5 . O aprofundamento do conhecimento da irrigação medular deve alterar e subsidiar a criação de novas estratégias de prevenção de isquemia medular durante a cirurgia aórtica e outros procedimentos neurocirúrgicos. Boll et al. analisaram 100 exames de pacientes portadores de neoplasia pancreática e aplicaram um algoritmo gráfico modificado de reconstrução de imagens de vasos cerebrais. A AKA foi visualizada em todos os casos, mas não podemos estabelecer se a alta detecção foi devida à ausência de doença aórtica ou ao diferente método de processamento de imagens 12 . 

 A identificação da AKA na literatura, por meio de tomografia computadorizada, é possível em torno de 70% dos casos analisados 1 ^,^ 6 ^,^ 8 ^-^ 12 , sendo que a causa da não identificação no restante não é clara. Foi sugerida a influência da aterosclerose, trombo mural, dissecção e massa corporal 3 ^,^ 5 . 

 O protocolo de imagem utilizado foi o mais próximo da angiotomografia habitual, de modo que representa o exame feito rotineiramente em muitos centros médicos. Observou-se com critério a minimização da dose de radiação para diminuir os riscos 13 . 

**Figura 4:**
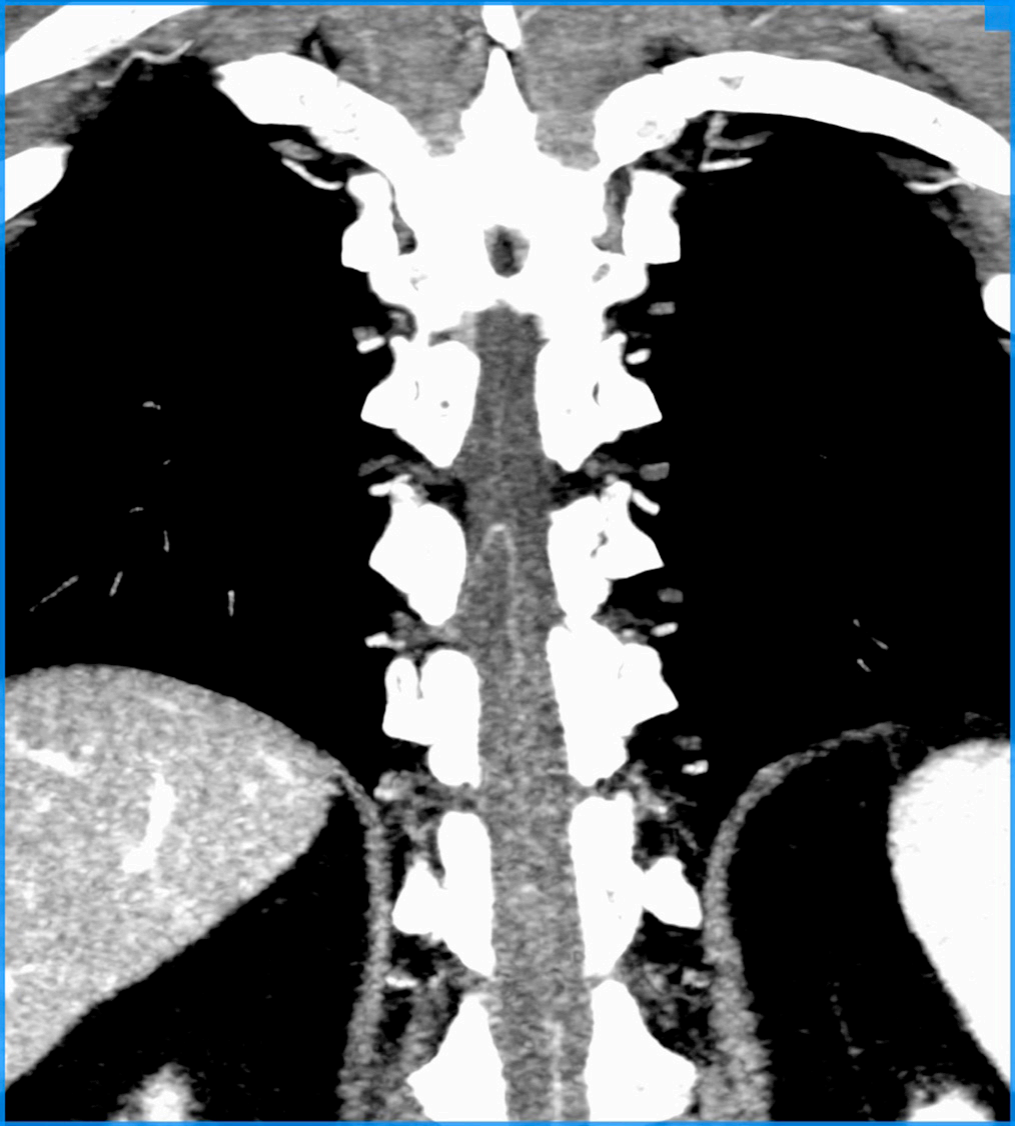
Resultado final da metodologia aplicada de identificação da AKA, em corte oblíquo no espaço medular de angiotomografia, demonstrando AKA visualizada.

 A angiotomografia, por ser um método baseado nos raios X, apresenta artefatos próximos de estruturas muito densas, como ossos. Os vasos estudados estão circundados por um arcabouço ósseo, porém o uso de equipamentos modernos permite a minimização desse problema 14 ^-^ 16 ( Figura 4 ). 

 Todas as variáveis de comorbidades e fatores de risco para aterosclerose testadas apresentaram diminuição na identificação da AKA. No grupo de pacientes fumantes, ex-fumantes e hipertensos, a chance de não identificação da AKA teve significância estatística. Os pacientes em que a AKA não pôde ser demonstrada apresentaram maior incidência de síndrome metabólica (31,1%), ao passo que, naqueles em que a AKA pôde ser avaliada, o índice de síndrome metabólica foi mais baixo (15,7%). Esse fato pode ser consequência dessa síndrome estar relacionada a danos vasculares ateroscleróticos mais avançados 17 , aumentando em 2,42 vezes (p = 0,054) a chance de não identificação da AKA na comparação com os que não apresentam síndrome metabólica. Todas as variáveis binárias de doenças da aorta testadas (trombo mural, dissecção e/ou trombo, aneurismas com e sem disseção) apresentaram redução na identificação da AKA com significância estatística, provavelmente por alteração do fluxo sanguíneo na aorta e seus ramos, oclusão do óstio das artérias intercostais, tortuosidade das artérias por distorção anatômica, oclusão pelo *flap* da dissecção, por placa aterosclerótica ou por trombo mural. Quando a AKA não é identificada, ela pode estar ocluída, pode não existir ou pode ser fina demais para detecção pelo método utilizado. 

 Foram identificadas variáveis clínicas de significância para identificação da AKA, e que permitiriam a construção de modelo matemático para predição de resultado. A ausência de um método padrão-ouro com baixo risco para comparação de métodos é uma limitação deste estudo. A estipulação de um valor limite de radiação aceitável também limitou a qualidade da imagem adquirida; porém, o método utilizado necessitou, em média, de 12 mSv/(mGy·cm), dose considerada segura, reprodutível 18 ^,^ 19 e que propicia cobertura satisfatória da aorta. 

## Conclusão

 Com as covariáveis etnia, IMC, tabagismo, hipertensão arterial e dislipidemia, foi possível criar modelo matemático de predição de identificação da AKA. 

## References

[1] Melissano G, Bertoglio L, Civelli V (2009). Demonstration of the Adamkiewicz artery by multidetector computed tomography angiography analysed with the open-source software OsiriX. Eur J Vasc Endovasc Surg.

[2] Amato ACM, Stolf NAG (2015). Anatomy of spinal blood supply. J Vasc Bras.

[3] Amato ACM, Parga JR, Stolf NAG (2017). Predictors of Adamkiewicz artery and anterior spinal artery detection through computerized tomographic angiography. SAGE Open Med.

[4] Melissano G, Civilini E, Bertoglio L, Calliari F, Amato ACM, Chiesa R (2010). Angio-CT imaging of the spinal cord vascularisation: a pictorial essay. Eur J Vasc Endovasc Surg.

[5] Amato ACM, Parga JR, Stolf NAG (2017). Influential factors on the evaluation of Adamkiewicz artery using a 320-detector row computed tomography device. Ann Vasc Surg.

[6] Ou P, Schmit P, Layouss W, Sidi D, Bonnet D, Brunelle F (2007). CT angiography of the artery of Adamkiewicz with 64-section technology: first experience in children. AJNR Am J Neuroradiol.

[7] Hiratzka LF, Bakris GL, Beckman JA (2010). 2010 ACCF/AHA/AATS/ACR/ASA/SCA/SCAI/SIR/STS/SVM guidelines for the diagnosis and management of patients with thoracic aortic disease - executive summary: a report of the american college of cardiology foundation/american heart association task force on practice guidelines, american association for thoracic surgery, american college of radiology, american stroke association, society of cardiovascular anesthesiologists, society for cardiovascular angiography and interventions, society of interventional radiology, society of thoracic surgeons, and society for vascular medicine. Anesth Analg.

[8] Sposito AC, Caramelli B, Fonseca FAH (2007). IV brazilian guideline for dyslipidemia and atherosclerosis prevention: department of atherosclerosis of Brazilian Society of Cardiology. Arq Bras Cardiol.

[9] Gao L, Wang L, Su B, Wang P, Ye J, Shen H (2013). The vascular supply to the spinal cord and its relationship to anterior spine surgical approaches. Spine J.

[10] Forbes G, Nichols DA, Jack CR (1988). Complications of spinal cord arteriography: prospective assessment of risk for diagnostic procedures. Radiology.

[11] Savader SJ, Williams GM, Trerotola SO (1993). Preoperative spinal artery localization and its relationship to postoperative neurologic complications. Radiology.

[12] Boll DT, Bulow H, Blackham KA, Aschoff AJ, Schmitz BL (2006). MDCT angiography of the spinal vasculature and the artery of Adamkiewicz. AJR Am J Roentgenol.

[13] Sodickson A, Baeyens PF, Andriole KP (2009). Recurrent CT, cumulative radiation exposure, and associated radiation-induced cancer risks from CT of adults. Radiology.

[14] Joseph PM, Ruth C (1997). A method for simultaneous correction of spectrum hardening artifacts in CT images containing both bone and iodine. Med Phys.

[15] Joseph PM, Spital RD (1978). A method for correcting bone induced artifacts in computed tomography scanners. J Comput Assist Tomogr.

[16] Goodenough DJ, Weaver KE, Costaridou H, Eerdmans H, Huysmans P (1986). A new software correction approach to volume averaging artifacts in CT. Comput Radiol.

[17] Olijhoek JK, van der Graaf Y, Banga JD (2004). The metabolic syndrome is associated with advanced vascular damage in patients with coronary heart disease, stroke, peripheral arterial disease or abdominal aortic aneurysm. Eur Heart J.

[18] Huda W, Ogden KM, Khorasani MR (2008). Converting dose-length product to effective dose at CT. Radiology.

[19] Van Unnik JG, Broerse JJ, Geleijns J, Jansen JT, Zoetelief J, Zweers D (1997). Survey of CT techniques and absorbed dose in various dutch hospitals. Br J Radiol.

