# Supplementary material for: Desenvolvimento de modelo clínico para predição da possibilidade de identificação da artéria de Adamkiewicz por angiotomografia
Source: J Vasc Bras. 2018 Jan-Mar;17(1):19–25. [Article in Portuguese] doi: 10.1590/1677-5449.006317 (PMC5990262; doi:10.1590/1677-5449.006317)
Supplement: Translation

# Translation: Development of a clinical model to predict the likelihood of identification of the Adamkiewicz artery by angiotomography

**Authors:** Alexandre Campos Moraes Amato, José Rodrigues Parga, Noedir Antônio Groppo Stolf

**Affiliations:** 1 Instituto de Medicina Avançada – AMATO, Cirurgia Vascular e Endovascular, São Paulo, SP, Brasil.; 2 Universidade de Santo Amaro – UNISA, Disciplina de Cirurgia Vascular, São Paulo, SP, Brasil.; 3 Universidade de São Paulo – USP, Departamento de Radiologia, Faculdade de Medicina, Hospital das Clínicas, Instituto do Coração, São Paulo, SP, Brasil.; 4 Universidade de São Paulo – USP, Cirurgia Torácica e Cardiovascular, São Paulo, SP, Brasil.

**Keywords:** spinal marrow, spinal column, aorta, Adamkiewicz

## Abstract

**Background:**

There are clinically important morphological differences in the Adamkiewicz artery (AKA) between populations that do and do not have aortic disease and they have an influence on the neuroischemic complications involving the spinal cord during surgical operations. It is not yet known whether clinical parameters correlate with the predictability of identification of the artery using angiotomography.

**Objective:**

To develop a mathematical model that by correlating clinical parameters with atherosclerosis enables prediction of the probability of identification of the AKA in patients examined with angiotomography.

**Method:**

This is a cross-sectional, observational study using a patient database and image bank. A multivariate statistical analysis was conducted and a logit mathematical model was constructed to predict AKA identification. Significant variables were used to build a formula for calculation of the probability of identification. This model was calibrated and its power of discrimination was assessed using receiver operating characteristic (ROC) curves. Selection of explanatory variables was based on largest area under the ROC curve (p = 0.041) and combined significance of variables.

**Results:**

A total of 110 cases were analyzed (54.5% were male, mean age was 60.97 years, and ethnicity coefficients were white -2.471, brown -1.297, and black -0.971) and the AKA was identified in 60.9%. Body mass index: 27.06 ± 0.98 (coef. -0.101); smokers: 55.5% (coef. -1.614/-1.439); diabetes: 13.6%; hypertension: 65.5% (coef. -1.469); dyslipidemia: 58.2%; aortic aneurysm: 38.2%; aortic dissection: 12.7%; and mural thrombus: 24.5%. The constant was 6.262. The formula for calculating the probability of detection is as follows: ${\left(e^{-\left(Coef.\;Etnicity+\left(Coef.BMI\times BMI\right)+Coef.smo\ker+Coef.SAH+Coef.dyslip+Cons\tan t\right)}+1\right)}^{-1}$ . The prediction model was constructed and made available at: https://vascular.pro/aka-model  .

**Conclusions:**

Using the covariates ethnicity, body mass index, smoking, arterial hypertension, and dyslipidemia, it proved possible to create a mathematical model for predicting identification of the AKA with a combined significance of nine coefficients (p = 0.042).

## Introduction

 Studying morphological differences of the Adamkiewicz artery (AKA) between populations with and without diseases is of clinical importance, because they can influence neuroischemic complications of the spinal cord during surgical operations. The findings could also help to establish the importance of preserving this artery in aortic and neurological surgery, reducing the risk of spinal cord ischemia. 1 ^-^ 3

 The intercostal and lumbar arteries that feed the spinal cord originate from the aorta. The intercostal and lumbar arteries divide three times before reaching the spinal cord. The last bifurcation of the spinal branch is constant for anterior and posterior supply of the vertebral canal, at some levels only. Generally, one of the anterior radicular arteries is dominant over the others, in terms of caliber, and is called the great radicular artery or the AKA. 2

 Knowledge of the blood supply to the spinal cord is important when planning treatment for aortic diseases, but the vasculature is complex and difficult to study because of the small caliber of the arteries involved, which form an intricate three-dimensional network with a large degree of anatomic variability 2 ^,^ 4 ( Figure 1 ). The fact that there is no consensus on a gold standard imaging exam also makes it difficult to compare existing imaging methods. 

**Figure 1:** Illustration of the anatomy of the spinal blood supply, after removal of the vertebral body.

 Differences in the AKA’s importance to spinal blood supply between different diseases, such as aneurysms, dissections, mural thrombi, and others, are also unknown, even though this is the population that is most likely to need surgical treatment and, consequently, most exposed to the risk of spinal cord ischemia. It has been demonstrated that aortic disease itself can change the appearance of the spinal blood supply. 5 While it is known which factors influence identification of the vessels of the spinal blood supply using angiotomography, their effects in combination are unknown. 

## Objective

 To construct a mathematical model using clinical variables to predict whether or not the AKA will be identifiable with angiotomography. 

## Method

### Patients

 We conducted a cross-sectional observational study of a database and archived images from a prior study 5 of patients with or without aortic disease who had been examined with angiotomography between October 2011 and July 2012. 

 During this period, the Instituto do Coração do Hospital das Clínicas da Faculdade de Medicina da Universidade de São Paulo conducted a total of 128 angiotomographies of the aorta in the diagnostic imaging department, using an Aquilion One scanner (Toshiba™, Medical Systems, Otawara, Japan). One hundred and ten of these examinations were considered eligible for the study, on the basis of the inclusion and exclusion criteria. 

 The inclusion criteria were as follows: patients who had undergone angiotomography of the aorta using an Aquilion One scanner and following a predefined protocol, irrespective of the reasons for ordering the examination; patients who accepted an aortic angiotomography as a supplementary test, in cases in which the treating physician had not ordered the examination. 

 The exclusion criteria employed were as follows: patients who had previously undergone surgery on the descending aorta; patients with rare diseases that could compromise the results because of collateral circulation (Takayasu’s disease, aortic coarction); paraplegic or tetraplegic patients; known allergy to radiology contrast; concurrent enrollment on another study that would interfere with the protocol; patients with intramural hematoma and penetrating aortic ulcer; age less than 25 years, to ensure sample homogeneity; technical failure of opacification of the aorta. 

 Routine outpatients angiotomographies were analyzed prospectively, using the open source software package OsiriX*®* (Pixmeo, Geneva, Switzerland). 6 Images were blindly analyzed; the evaluator was unaware of why the examination had been conducted and assessments of whether aortic disease was present were made in a second round of analyses of the images, during which the evaluator was unaware of whether or not the AKA had been identified. 

 Angiotomography examinations were conducted according to a preestablished routine: images were all acquired using the same Aquilion One, 320-detector scanner, with software for helical scanning with 80 to 160 detectors, following the protocol for arterial contrast studies with 100 to 110 mL of non-ionic contrast administered intravenously over 30 s with an injection pump at a velocity of 4 to 5 mL/s, with the trigger configured for a greater contrast concentration in the descending aorta and a threshold of 150 HU. The technical parameters of the angiotomographic method used follow the recommendations contained in aortic disease diagnosis and treatment guidelines. 7

 Patients were stratified according to the following characteristics: sex, age, ethnic group, weight, height, and body mass index (BMI). Presence or absence of the anterior spinal artery, presence and location of the AKA, and presence or absence of aortic disease, thrombus, and dissection were evaluated by a single, experienced observer directly on the highest quality raw DICOM images from the patient’s examination. Additionally, dichotomous risk factors related to aortic diseases were also assessed: diabetes mellitus, metabolic syndrome, hyperlipidemia, and arterial hypertension. The variable smoking was categorized as smoker, ex-smoker, or non-smoker. 

 Sociodemographic data were obtained from the patients’ medical records and directly from patients and their relatives, as follows: age, sex, and ethnicity. Ethnicity was defined according to the skin color reported by the participant, i.e. self-assessed. Self-reported multiracial variations were coded as brown skin. Patients were also told they could ignore this question. 

 Data were collected when taking patients’ histories, from medical records, and by telephone from relatives when necessary. The following parameters were evaluated: weight, height, and BMI. Weight and height were measured using an anthropometric balance; BMI was calculated with the formula: weight (in kilograms) divided by the square of height (in meters). Smoking: patients were defined as current smokers if they had smoked one or more cigarettes during the preceding 30 days and as ex-smokers if they had ever smoked for more than 30 days. Diabetes mellitus (DM): patients were classified as diabetics if they had been taking oral hypoglycemic drugs and/or insulin, or had two fasting glycemia results ≥ 126 mg/dL. Systemic arterial hypertension (SAH): patients were considered hypertensive if they were taking antihypertensives and/or had systolic arterial blood pressure (SBP) greater than 140 mmHg and/or diastolic arterial blood pressure (DBP) greater than 90 mmHg. Dyslipidemia: patients were classified as having dyslipidemia if they were taking antihyperlipidemics and/or had elevated low density lipoprotein cholesterol (LDL-C ≥ 160 mg/dL) and/or elevated triglycerides (TG ≥ 150 mg/dL) and/or high density lipoprotein cholesterol (HDL-C) < 40 mg/dL for men and < 50 mg/dL for women. 8 Metabolic syndrome: diagnoses of metabolic syndrome were made according to International Diabetes Federation criteria. Presence of abdominal obesity is an essential criterion, combined with two or more of the following criteria: triglycerides ≥ 150 mg/dL; HDL-C < 40 mg/dL for men and < 50 mg/dL for women; SBP ≥ 130 mmHg and/or DBP ≥ 85 mmHg, or treatment for hypertension; fasting glycemia ≥ 100 mg/dL or treatment for diabetes mellitus. 8

 The method used to identify AKA has been used by the author, described and validated in a previous publication, 3 ^,^ 6 and an explanatory video illustrating the technique is available at http://vascular.cc/aka.html  . 

### Statistical analysis

 The data obtained from patient histories and medical records were input on a secure online database (GoogleDocs®). After verification of the consistency of data, descriptive analyses were conducted for each group of patients. 

 Multivariate statistical analysis of data was conducted using Wizard 1.9.7 software (Evan Miller, Chicago, US). Models were defined as good if the area under the receiver operating characteristic (ROC) curve was greater than 0.7 and as excellent if the area under the ROC curve exceeded 0.85. The logit model was chosen since the outcome was binary and significance was defined as p < 0.05. Once the model had been constructed, a formula was derived to predict the outcome variable. The prediction formula calculates the expected value of the outcome (identification or failure to identify the AKA) based on the initial variables selected as meeting the criterion of area under the ROC curve greater than 0.7. 

 Considering the entire sample, Table 1 lists the variables collected and the preliminary analyses of the database that have been published previously. 3 ^,^ 5 The outcome selected was identification or failure to identify the AKA using the method proposed. The initial explanatory co-variables were chosen on the basis of prior knowledge of the influence of body mass, arterial disease, and atherosclerosis. Covariates with coefficients significantly different from zero to the 10% level were maintained. Thus, ethnicity, smoking, BMI, hypertension, and dyslipidemia were chosen to construct the predictive model ( Table 2 ). Selection of explanatory variables was guided by largest area under the ROC curve ( Figure 2 A) and by the combined significance of the variables ( Figure 2 B), so that a balance was achieved between the number of explanatory variables, without excessive variables, and the greatest influence on area under the ROC curve. 

**Table 1:** Sociodemographic data on the study population.

**Characteristic**		**N = 110**
Sex	Male	60 (54.5%)
	Female	50 (45.5%)
Age	Mean	60.97 ± 2.34
BMI	Mean	27.06 ± 0.98
Ethnicity (skin color)	Yellow	6 (6.8%%)
	White	55 (62.5%)
	Brown	21 (23.9%)
	Black	6 (6.8%)
AKA	Yes No	67 (60.9%) 43 (39.1%)
Smoker	Yes No Ex-smoker	61 (55.5%) 32 (29.1%) 17 (15.5%)
Diabetes	Yes No	15 (13.6%) 95 (86.4%)
SAH	Yes No	72 (65.5%) 38 (34.5%)
Dyslipidemia	Yes No	46 (58.2%) 64 (41.8%)
Metabolic syndrome	Yes No	25 (22.7%) 85 (77.3%)
Aortic aneurysm	Yes No	42 (38.2%) 68 (61.8%)
Dissection of the aorta	Yes No	14 (12.7%) 96 (87.3%)
Mural thrombus	Yes No	27 (24.5%) 83 (75.5%)

AKA, Adamkiewicz artery; SAH, systemic arterial hypertension; BMI, body mass index.

**Table 2:** Significance of variables in the model studied and coefficients for the influence of variables on the outcome.

**Explanatory variable**	**Coefficient**	**Standard error**	**p**	**Significance**
Ethnicity (skin color)			0.0677	*
White	-2.471	(1.264)	0.0505	*
Brown	-1.297	(1.329)	0.3291	
Black	-0.971	(1.597)	0.5433	
BMI	-0.101	(0.055)	0.0664	*
Smoker			0.0196	**
Yes	-1.614	(0.801)	0.0439	**
Ex-smoker	-1.439	(0.595)	0.0156	**
SAH	-1.469	(0.604)	0.0150	**
Dyslipidemia	0.97	(0.555)	0.0806	*
Constant	6.262	(2.018)	0.0019	***

* Coefficient significantly different from zero to the 10% level;

** Coefficient significantly different from zero to the 5% level;

*** Coefficient significantly different from zero to the 1% level.

SAH, systemic arterial hypertension; BMI, body mass index.

**Figure 2:** (a) Receiver operating characteristic (ROC) curve. The greater the area under the curve, the better the fit (1.0 = perfect; 0.5 = random). Area under the curve = 0.8039; (b) T distribution used to test the hypothesis centered on the point of estimation, with test of significance for nine selected covariates, with a result of p = 0.0415.

 This investigation was conducted in accordance with the principles set out in the Helsinki Declaration. The study was approved by the Scientific Commission at the Instituto do Coração (InCor), Universidade de São Paulo, and the Research Ethics Commission at the HC-FMUSP on 05/05/2010, under reference number 0089/10. It is registered on the Brazilian national human research ethics register (SISNEP) maintained by the National Research Ethics Commission (CONEP - Comissão Nacional de Ética em Pesquisa), under number 0088.0.015.000-10. All patients or their guardians were given explanations about the objectives of the study, agreed to participation, and signed free and informed consent forms, which included the current data analysis in the terms of permission. 

### Results

The formula for calculating the probability of detecting the AKA

${\left(e^{-\left(Coef.\;Etnicity+\left(Coef.BMI\times BMI\right)+Coef.smo\ker+Coef.SAH+Coef.dyslip+Cons\tan t\right)}+1\right)}^{-1}$ enabled construction of the prediction model, which was exported to an excel spreadsheet ( Figure 3 ). Table 2 lists the constant and the coefficients for the influence of the variables selected for calculation of the outcome. The model was formatted and sent to an online service and then published for general use at the web address https://vascular.pro/aka-model  . The variables can be changed in real-time and calculate the probability of identification of the AKA. 

**Figure 3:** Simplified appearance of the model for prediction of identification of the AKA on a basic electronic spreadsheet. Interface for data collection for calculating the probability of the outcome.

## Discussion

 Spinal cord ischemia is a possible complication of aortic surgery and, while rare, it is devastating and merits study. 9 In vivo examination of the AKA is difficult because the method considered to be the gold standard, angiography, currently has unacceptably high rates of serious complications: 8.2% for localized complications, 3.7% for non-neurological complications, and 2.2% for neurological complications, 10 and does not achieve detection in 100% of cases (without aortic disease: 68%; 10 with aortic disease: 60%). 11

 The angiotomographic characteristics of the AKA in populations with and without aortic disease have been studied and reported in an earlier publication by the author. 3 ^,^ 5 Improved knowledge of the spinal blood supply should affect and provide a foundation for creation of new strategies for prevention of spinal cord ischemia during aortic surgery and other neurological surgical procedures. Boll et al. analyzed 100 examinations of patients with pancreatic cancer and applied a modified version of a graphical algorithm for reconstruction of images of cerebral vessels. The AKA was identified in all cases, but it is unclear whether the high rate of detection was because of the absence of aortic disease or the different method used to process the images. 12

 According to the literature, it is possible to identify the AKA using computed tomography in around 70% of cases analyzed, 1 ^,^ 6 ^,^ 8 ^-^ 12 although the cause of non-identification in the remaining cases is unclear. Influence from atherosclerosis, mural thrombus, dissection, and body mass has been suggested. 3 ^,^ 5

 The imaging protocol used was as close as possible to habitual angiotomography protocols, so that it represented the routine examinations conducted at many medical centers. Care was taken to minimize the radiation dose to reduce risks. 13

 Since angiotomography is a method based on X-rays, it suffers from artifacts close to very dense structures, such as bones. The vessels studied are surrounded by a bony framework, but modern equipment allows this problem to be reduced to a minimum 14 ^-^ 16 ( Figure 4 ). 

**Figure 4:** Final result of the methodology employed for identification of the AKA, angular angiotomography slice of the spinal space, showing the AKA.

 All of the variables tested representing comorbidities and risk factors for atherosclerosis were associated with lower rates of AKA identification. In the subset of smokers, ex-smokers, and hypertensive patients, the likelihood of non-identification of the AKA was statistically significant. Patients in whom the AKA could not be identified had a higher incidence of metabolic syndrome (31.1%), compared with those in whom the AKA could be evaluated, who had a lower rate of metabolic syndrome (15.7%). This fact may be a consequence of this syndrome being related to more advanced atherosclerotic vascular damage, 17 increasing the likelihood of non-identification of the AKA by 2.42 times (p = 0.054) in comparison with those who did not have metabolic syndrome. All of the binary aortic disease variables tested (mural thrombus, dissection and/or thrombus, and aneurysms with and without dissection) were associated with reductions in identification of AKA with statistical significance, probably because of abnormal blood flow through the aorta and its branches, occlusion of the ostia of intercostal arteries, tortuosity of arteries secondary to anatomic distortion, and/or occlusion by dissection flap, atherosclerotic plaque, or mural thrombus. When the AKA is not found, it could be occluded, it may not exist, or it may be too narrow to be detected by the method used. 

 Clinical variables were identified that have significance with relation to identification of the AKA, enabling a mathematical model for prediction of the result to be constructed. The absence of a low-risk gold-standard method for comparison of methods is one factor limiting this study. Setting a maximum limit for acceptable radiation dosage also limited the quality of the images acquired. However, the method employed required a mean of 12 mSv/(mGy·cm), which is considered a safe dose, is reproducible, 18 ^,^ 19 and provides satisfactory coverage of the aorta. 

## Conclusions

 It proved possible to use the covariates ethnicity, BMI, smoking, arterial hypertension, and dyslipidemia to create a mathematical model to predict identification of the AKA.
